# The Effect of Superparamagnetic Iron Oxide with iRGD Peptide on the Labeling of Pancreatic Cancer Cells *In Vitro*: A Preliminary Study

**DOI:** 10.1155/2014/852352

**Published:** 2014-05-19

**Authors:** Hou Dong Zuo, Wei Wu Yao, Tian Wu Chen, Jiang Zhu, Juan Juan Zhang, Yu Pu, Gang Liu, Xiao Ming Zhang

**Affiliations:** ^1^Sichuan Key Laboratory of Medical Imaging, Department of Radiology, Affiliated Hospital of North Sichuan Medical College, Nanchong, Sichuan 637000, China; ^2^Department of Radiology, Shanghai Jiao Tong University Affiliated Sixth People's Hospital, 600 Yishan Road, Shanghai 200233, China

## Abstract

The iRGD peptide loaded with iron oxide nanoparticles for tumor targeting and tissue penetration was developed for targeted tumor therapy and ultrasensitive MR imaging. Binding of iRGD, a tumor homing peptide, is mediated by integrins, which are widely expressed on the surface of cells. Several types of small molecular drugs and nanoparticles can be transfected into cells with the help of iRGD peptide. Thus, we postulate that SPIO nanoparticles, which have good biocompatibility, can also be transfected into cells using iRGD. Despite the many kinds of cell labeling studies that have been performed with SPIO nanoparticles and RGD peptide or its analogues, only a few have applied SPIO nanoparticles with iRGD peptide in pancreatic cancer cells. This paper reports our preliminary findings regarding the effect of iRGD peptide (CRGDK/RGPD/EC) combined with SPIO on the labeling of pancreatic cancer cells. The results suggest that SPIO with iRGD peptide can enhance the positive labeling rate of cells and the uptake of SPIO. Optimal functionalization was achieved with the appropriate concentration or concentration range of SPIO and iRGD peptide. This study describes a simple and economical protocol to label panc-1 cells using SPIO in combination with iRGD peptide and may provide a useful method to improve the sensitivity of pancreatic cancer imaging.

## 1. Introduction

The development of metallic or inorganic nanoparticles for disease and cancer diagnostic imaging has progressed rapidly in recent years due to their unique physical characteristics, favorable biocompatibility, and specific targeting capabilities [1–5]. Among them, superparamagnetic iron oxide (SPIO) nanoparticles have a great potential for basic and clinical application due to their several advantages, such as guided transport or distribution under an external magnetic field* in vivo* and T2-type magnetic resonance imaging contrast enhancement [3, 4, 6–10]. In particular, negative contrast agents (e.g., SPIO) for molecular imaging that decrease the T2 and T2* relaxation times of tissues and provide higher sensitivity for MRI are advantageous because they have strong contrast effects, conveniently controlled magnetic characteristics, and improved biodegradability [11]. Cationic surfaces facilitate cellular internalization [12]. Currently, there is a huge research impetus in MR diagnostic imaging to develop hybrid SPIO nanoparticles integrated with multiple imaging detection components [3, 5, 6, 8, 13]. To date, SPIOs modified or coated with dextran, polystyrene, polyethylene glycol (PEG), and polylysine (PLL) have been studied and applied [11, 13–15].

Integrins, which consist of an *α* and a *β* subunit, are a family of heterodimeric glycoprotein receptors on the cell surface which mediate and diversify biological communication involving cell adhesion and signal transduction [16]. Currently, 24 integrin subtypes have been reported [17, 18], while *α*v*β*3 integrin that is overexpressed on tumor cells is one of the most prominent receptors involved in tumor growth, invasiveness, and metastasis [16, 19–22]. The *α*v integrins and neuropilin-1 are expressed on pancreatic cancer cells, including the panc-1 cell line [23–29]. In addition, *α*v*β*3 integrin can be targeted by peptides with a short amino acid sequence containing Arg-Gly-Asp (RGD) [3, 22, 30–32]. The iRGD (CRGDK/RGPD/EC) peptide is a newly identified type of tumor-penetrating peptide, which was discovered by phage display. The peptide can increase the permeability of tumor cells, mediate cellular internalization and extravasation, and enhance deep tissue penetration to improve the imaging sensitivity and therapeutic efficacy mediated by integrins and neuropilin-1 (NRP-1) receptors [3, 23, 30]. The iRGD peptide plays a part in targeting tumor cells using a similar procedure to conventional RGD peptides [3, 30]. Recently, the application of RGD peptide with SPIO has been an active focus of research. Zhang et al. [31] evaluated RGD-USPIO uptake in cultured human umbilical vein endothelial cells (HUVECs)* in vitro* by MRI.

Here, we propose and investigate a new strategy for integrin targeting and tumor diagnostic imaging based on iRGD peptide. We aim to (1) study the effect of SPIO with iRGD peptide on the labeling of pancreatic cancer cells* in vitro* and (2) find a new and useful modality for pancreatic cancer diagnostic imaging. To our knowledge, there have been no studies investigating the biophysical properties of SPIO with iRGD peptide for cellular MR imaging. Therefore, this study is novel, and previous studies suggest that it is feasible. The intracellular uptake of Fe, the viability of labeled cells, and their MRI signal intensity (SI) changes were assessed. The results suggest that our modality may be useful for alternative cell labeling and pancreatic cancer diagnostic imaging.

## 2. Materials and Methods

### 2.1. Chemicals, Reagents, and Experimental Instruments

The human pancreatic cancer cell line (panc-1) was provided by Sichuan University. The following other materials and instruments were used: iRGD peptide, SPIO (Resovist, SHU555A, SCHERING Company, Germany), polylysine, fetal bovine serum, RPMI-1640 solution, trypsin, a 3.0 T MR scanner (Discovery MR 750; GE Medical Systems, Milwaukee, WI), an inverted microscope (TS100-F), an oven (DGX-9143), and an atomic absorption spectrophotometer (AAS, SpectrAA 220FS, 12°C, 63% humidity).

### 2.2. The Concentration of Reagents and Solutions

The nutrient solution consisted of 85% RPMI-1640 solution and 15% fetal bovine serum. SPIO mixed solution had 840 *μ*g iron ion per mL. The following solutions were used: 10% iRGD peptide solution, 0.5% neutral red solution, PBS buffer solution (0.1 mol/L, pH 7.4), and 0.25% trypsin. The iron ion concentration of SPIO and the PLL mixture solution was 840 *μ*g per mL. Perl's staining solution was composed of 2% potassium ferrocyanide solution and 3% diluted hydrochloric acid mixed in an equal volume.

### 2.3. Cell Culture and Treatment

The human pancreatic cancer cells (1 × 10^6^ cells/well, 9.6 cm^2^ per well) were cultured for 24 hours. Then, the cells were washed with phosphate buffered saline (PBS) three times. Afterwards, the SPIO and iRGD peptide solutions were coadministered. The total solution volume was 2 mL per well. The iron ion concentrations were 8.4 *μ*g/mL, 12.6 *μ*g/mL, 14.7 *μ*g/mL, 16.8 *μ*g/mL, and 21 *μ*g/mL. The iRGD peptide solution concentrations added were 0.25 *μ*g/mL, 0.5 *μ*g/mL, 0.75 *μ*g/mL, 1.0 *μ*g/mL, and 1.25 *μ*g/mL, respectively. Considering the 8.4 *μ*g/mL concentration as an example, 1980 *μ*L, 1980 *μ*L, 1975 *μ*L, 1970 *μ*L, 1965 *μ*L, 1960 *μ*L, and 1955 *μ*L of nutrient culture were first added to each of the 6 wells. Next, 20 *μ*L SPIO solution was added to each well followed by 0 *μ*L, 0 *μ*L, 5 *μ*L, 10 *μ*L, 15 *μ*L, 20 *μ*L, and 25 *μ*L iRGD peptide solution, respectively. As control, SPIO-PLL was added in another well with each SPIO concentration series. Next, the cells were incubated for 24 hours. After incubation, the culture medium was removed. The adherent cells were washed with PBS three times, trypsinized, washed with PBS, and centrifuged for 5 minutes at 1000 rpm. The total number of cells (1 × 10^6^) was determined using a counting chamber. Then, the cells were embedded in agar (1%) at room temperature for MRI examination.

### 2.4. Cell Viability

The viability of the cells cultured under different conditions was evaluated with 0.4% trypan blue dye solution. The percentage of nonviable or dead cells was determined by counting trypan blue-positive and trypan blue-negative cells in a counting chamber. The nonviable rate was calculated. The group with the highest concentration of iRGD peptide within each iron concentration series was analyzed.

### 2.5. MR Sequence, Parameters, and Measurement

MR T2 relaxometry of panc-1 cells in agar was performed using a fast recovery fast spin echo T2 weighted (FRFSE-T2W) sequence with a 32-channel head coil in a 3.0 T MR scanner (slice thickness: 2 mm; interslice space: 0–0.5 mm; TR: 2800.0 ms; TE: 90.3 ms; DFOV: 18.0 × 18.0 cm; reconstruction matrix: 512 × 256). All the original images were transferred to an ADW4.4 workstation, and the best images were selected. Identification of 25 mm^2^ regions of interest (ROI) was performed in triplicate, and the mean values were adopted. The signal intensity reduction value (Δ*S*) of every sample (including the SPIO-PLL group) relative to the control group was measured within the same SPIO concentration. The signal intensity changes (Δ*S*′) of every sample (including the SPIO control group) were also measured relative to the PBS blank group with the same SPIO concentration to analyze the appropriate concentration of iRGD to SPIO concentration. All the data were processed and measured on the ADW4.4 workstation.

### 2.6. Prussian Blue Staining

For Prussian blue staining, the cells were cultivated for 24 hours in 6-well plates on glass coverslips (1 × 10^6^ cells). After incubation, the labeled panc-1 cells were washed three times with PBS and subsequently fixed with 4% paraformaldehyde for 40 to 60 minutes. Then, the fixed cells were incubated with Perl's dye for 30 minutes and counterstained with 0.5% neutral red solution for 1 minute.

### 2.7. Inverted Microscope Observation and Transmission Electron Microscopy

The cells on glass coverslips were placed on slides, fixed, and then observed with an inverted microscope. The cells were routinely observed using 10x, 20x, and 40x magnification views. Photos were taken at random sites under 40x magnification. The positive labeled cells were counted under 4 magnification views (40x and 20x) randomly, and the positive labeled rate was calculated (positive labeled cell counts/total cell counts × 100%). The cells cultured with 12.6 *μ*g/mL iron ion and 1 *μ*g/mL iRGD peptide were selected for observation with a transmission electron microscope.

### 2.8. The Quantification of Iron Cell Content

After MRI scanning, the group with the best concentrations of SPIO and iRGD peptide was selected and the samples were treated with 15 mL aqua regia (*V*_HCL_ : *V*_HNO_3__ = 3 : 1) for the determination of the iron content by AAS.

### 2.9. Statistical Analysis

The continuous data were expressed as mean ± standard deviation (SD). Statistical analysis was conducted using a nonparametric Wilcoxon rank sum test. A 2-independent samples test was performed in the analysis of Δ*S*′ and cell viability. Statistical analysis was conducted with the SPSS16.0 software package for Windows (SPSS Institute, Chicago, IL, USA). An *α* level of 0.05 was used.

## 3. Results

### 3.1. Evaluation of Cell Viability

**Figure 1:**
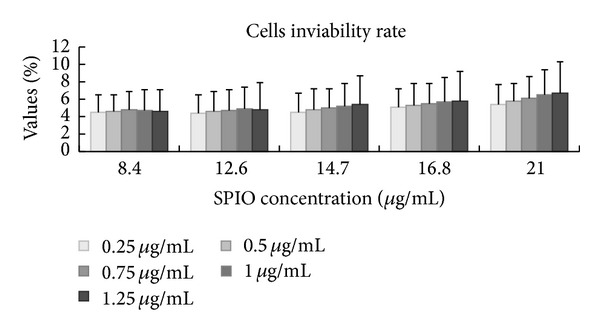
The rate of nonviable cells for different SPIO and iRGD peptide concentrations. As shown in the graph, trypan blue staining demonstrated that there was not a remarkable reduction in the viability of panc-1 cells after incubation with increasing concentrations of SPIO and iRGD peptide.

Trypan blue staining did not demonstrate reduced viability of panc-1 cells after incubation with increasing concentrations of SPIO and iRGD peptide, including the SPIO and SPIO-PLL control groups. There were no significant differences among the samples. For example, for the highest concentration of iron ions (21 *μ*g/mL) in this study, the percentages of nonviable cells for the different concentrations of the iRGD peptide samples were 5.4 ± 2.5%, 5.8 ± 3.1%, 6.1 ± 3.3%, 6.5 ± 3.4%, and 6.7 ± 3.6%, respectively. These values did not differ significantly from each other (Figure 1).

### 3.2. *In Vitro* MRI Evaluation of T2-W

**Figure 2:**
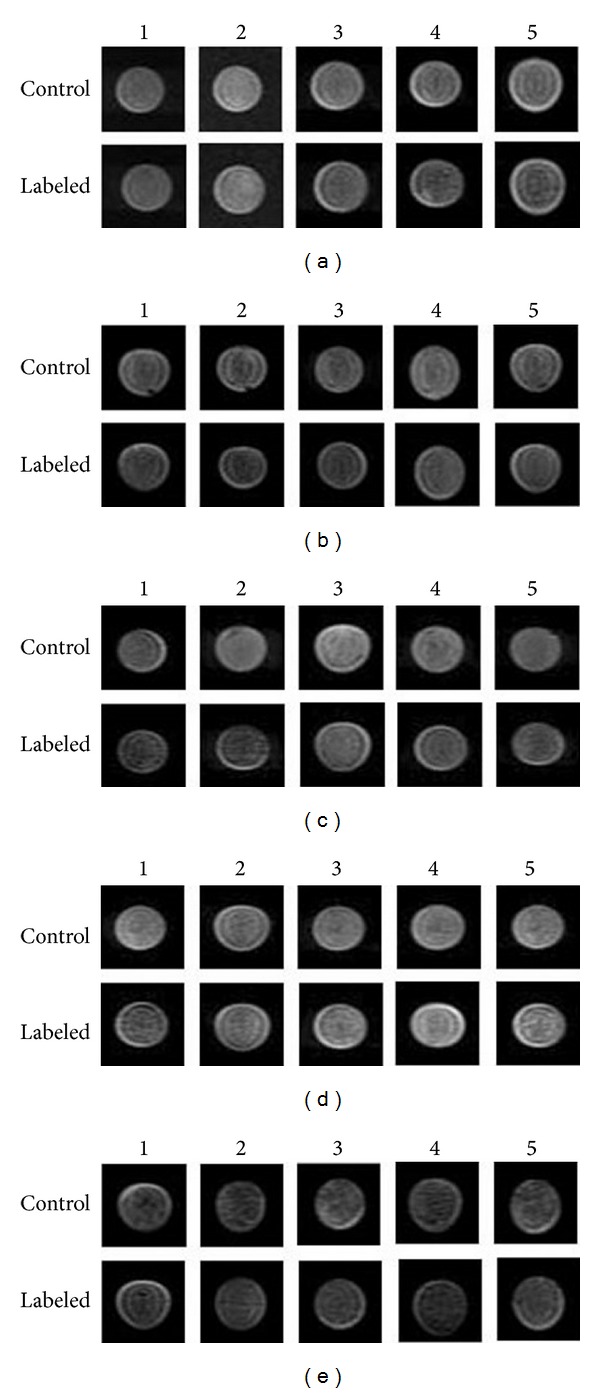
MR images of labeled cells in agar (1 × 10^6 ^cells) acquired using T2-weighted imaging with the FRFSE sequence. (a)–(e) indicate the iron ion concentration (from 8.4 to 21 *μ*g/mL, resp.). The numbers 1–5 represent the iRGD peptide concentration (from 0.25 to 1.25 *μ*g/mL, resp.).

**Figure 3:**
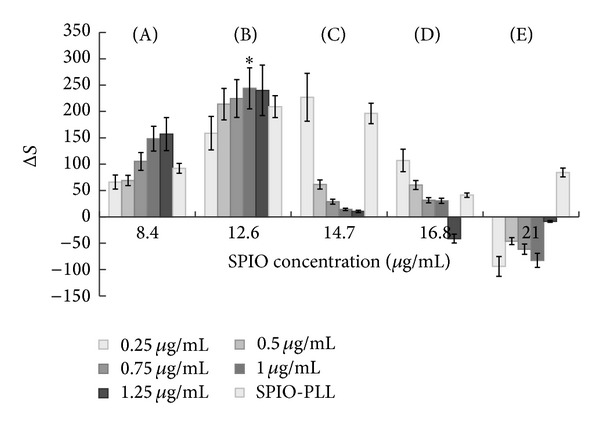
The evaluation of the mean signal reduction (Δ*S*) for different iron ion concentrations. (A)–(E) indicate 8.4–21 *μ*g/mL iron ion concentration. The SPIO-PLL group served as the control group. *The highest Δ*S* value (the best concentration match of iron ions and iRGD peptide).

When the concentration of iron was 8.4 *μ*g/mL, the signal intensity of the labeled cell samples decreased gradually with the increase in iRGD peptide concentration (Figures 2(a) and 3). The mean Δ*S* increased correspondingly. When the concentration reached 12.6 *μ*g/mL, the signal intensity first decreased and then increased with increasing concentrations of iRGD peptide. Therefore, the mean Δ*S* first increased and then decreased. The Δ*S* was the highest (243.89 ± 89.1) with 1 *μ*g/mL of iRGD peptide (Figures 2(b) and 3). When the iron concentrations were 14.7 *μ*g/mL and 16.8 *μ*g/mL, the signal intensity was enhanced with increasing concentration of iRGD. Consequently, the mean Δ*S* reduced gradually (Figures 2(c)-2(d) and 3). However, when the iron concentration was 21 *μ*g/mL, the signal intensities for different iRGD concentrations differed from each other and were higher than those of the control group (Figures 2(e) and 3). The mean Δ*S* of the SPIO-PLL group was slightly lower than that of groups with the appropriate iRGD peptide concentration and iron concentrations of 12.6 and 14.7 *μ*g/mL (Figure 3).

**Figure 4:**
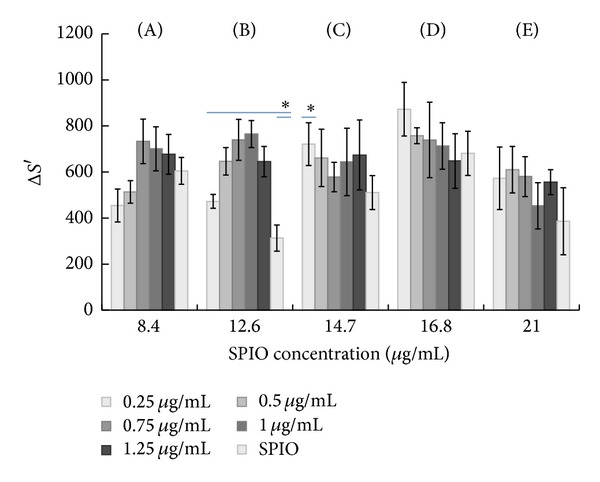
The evaluation of the mean signal reduction values (Δ*S*′) for different iron concentrations. For the iron concentration of 12.6 *μ*g/mL, a significant difference was found between the treatment and control groups (*P* < 0.05). For the iron concentration of 14.7 *μ*g/mL, a significant difference was only found between 0.25 *μ*g/mL iRGD and the control group. (A)–(E) indicate 8.4–21 *μ*g/mL iron ion concentration. The SPIO group served as the control group. *P < 0.05.

We also determined the appropriate concentration of iRGD peptide within the same series of iron ion concentrations. No significant difference in the mean Δ*S*′ was found between the treatment group and control groups (*P* > 0.05) for the 8.4 *μ*g/mL concentration of iron ions. However, when the concentration of iron ions was 12.6 *μ*g/mL, there was a significant difference between the treatment and control groups (*P* < 0.05). Only one group (0.25 *μ*g/mL iRGD) differed from the control group at the 14.7 *μ*g/mL concentration of iron ions. However, no significant difference was found for the other two iron ion concentrations. Therefore, our results suggest that the appropriate concentration of iRGD peptide was 0.25–1.25 *μ*g/mL for the 12.6 *μ*g/mL iron ion concentration and 0.25 *μ*g/mL for the 14.7 *μ*g/mL of iron ion concentration (Figure 4).

### 3.3. Microscope Observation, Characteristics of Prussian Blue Staining, and Transmission Electron Microscopy

**Figure 5:**
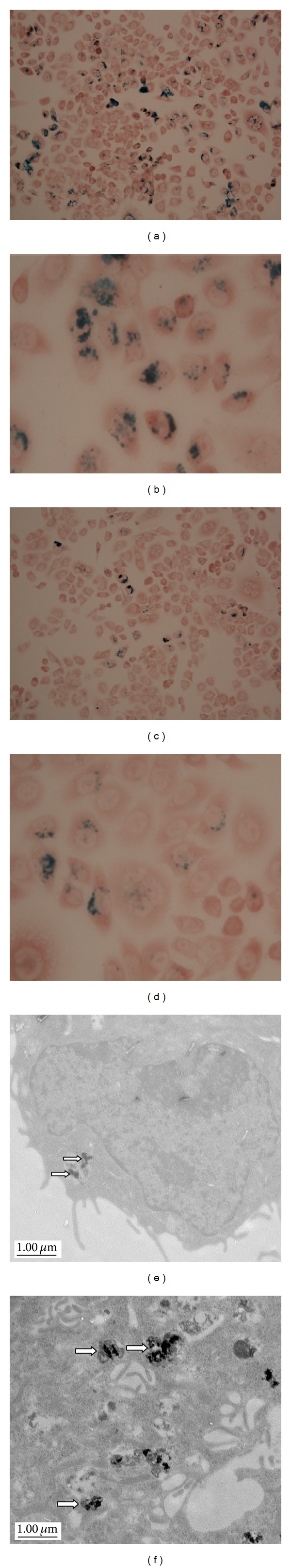
Prussian blue-stained and neutral red solution-counterstained panc-1 cells incubated with SPIO. (a), (b) The cells were incubated with 1 *μ*g/mL iRGD at an iron concentration of 12.6 *μ*g/mL ((a): 20x and (b): 40x). The cells of the control groups were incubated with SPIO without iRGD ((c): 20x and (d): 40x). Higher uptake of SPIO (blue-stained granules) with iRGD peptide compared with SPIO is clearly demonstrated (c, d). Electron microscopy of cells labeled with SPIO nanoparticles (e) and SPIO nanoparticles with iRGD peptide (f) (arrows).

**Figure 6:**
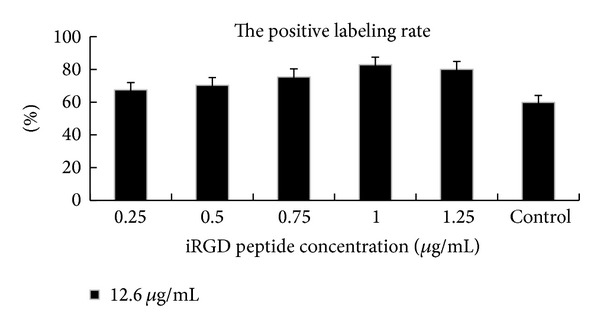
The evaluation of the positive labeled cells. The positive labeling rate of the control was lower than that of the iRGD treatment groups. Control denotes the control group (SPIO). The concentration of SPIO was 12.6 *μ*g/mL.

Cells treated with 12.6 *μ*g/mL concentration of SPIO and a range of iRGD peptide concentrations were selected for observation. The labeled cells were diamond or oval in shape under microscopic observation when treated with iron oxide and the range of iRGD. When the cells were incubated with 1 *μ*g/mL iRGD at an iron concentration of 12.6 *μ*g/mL, the positive labeling rate of the treatment group was enhanced relative to the control group (Figure 6). The morphology of the cells was good. Speckled, granular, and patchy blue-stained particles were observed in the cytoplasm, cell nucleus, and cell membrane (Figure 5). The granules were mostly in the cytoplasm around the nuclei. The distribution of the intracellular Fe granules observed under transmission electron microscope is shown in Figures 5(e)-5(f).

The positive labeling rate after treatment was not different than that of the control group with 12.6 *μ*g/mL SPIO (Figure 6). The iron content determination was confirmed by AAS analysis. The mean iron content (pg/cell) of each treatment sample with increasing concentration of iRGD peptide was 2.42 pg, 5.61 pg, 8.12 pg, 10.74 pg, and 13.20 pg, respectively, which was higher than that of the control sample (0.94 pg/cell).

## 4. Discussion

In the present study, the ability of SPIO combined with iRGD peptide to specifically bind to the *α*v*β*3 integrin on tumor cells was investigated* in vitro*. In contrast to previous studies of molecular MR probes coated or modified with dextran or peptides [33, 34], we explored a novel method of coadministration of SPIO and iRGD peptide that may be useful in the enhancement of tumor imaging. Our results suggest that this method was also effective in labeling cells for MR molecular imaging.

Imaging of pancreatic cancer cells using MRI provides good spatial resolution, resulting in exquisite dynamic information and anatomical contrast, while the kinetics of SPIO distribution in tumor xenografts can be monitored with noninvasive modalities [1, 35]. In recent years, T2 and T2* sequence-mediated MR imaging with SPIO nanoparticles has become a favorable technique that may be available in the clinic. SPIO is a kind of negative contrast that can reduce T2 and T2* values and has increased sensitivity for MRI due to susceptibility effects [31]. Therefore, intracellular iron has strong T2 and T2* effects, and the high reduction in signal is related to the increase in iron oxide uptake by the cells. Human pancreatic cancer cells can be labeled with SPIO and induce tumor xenografts in nude mice after injection. Our results have demonstrated the efficient nanoparticle uptake capabilities of cells.

To optimize MR imaging* in vitro* and maximize the reliability of the results, it is important that (i) the labeling is reproducible, (ii) the function and viability of labeled cells are retained, and (iii) the process does not affect the target cells [36]. Therefore, we analyzed the cell viability at the beginning of the study through trypan blue staining with different concentrations of iron oxide and iRGD peptide and demonstrated the feasibility of our method.

Several kinds of amino acids have been used to enhance the uptake of SPIO nanoparticles, and the use of polylysine (PLL) has frequently proven to be feasible and efficient [1]. However, Kobukai et al. demonstrated that the use of PLL may be a double-edged sword; the compound can be either safe or toxic depending on the concentration used [37]. Another group suggested that PLL may trigger an inflammatory response by releasing the proinflammatory cytokine, tumor necrosis factor (TNF) [38].

Here, we investigated a new peptide (iRGD peptide) that has a similar function to PLL. The cyclic peptides containing the RGD sequence home to the *α*v*β*3 integrin specifically and have been applied widely to integrin targeting on cancer pathology, molecular imaging, and drug delivery [3, 30, 31, 34]. This kind of disulfide-based cyclic RGD peptide, called iRGD (CRGDK/RGPD/EC), can interact with both integrin and neuropilin-1 receptors to mediate cellular internalization to improve imaging sensitivity [3]. Sugahara et al. [30]. reported that iron-oxide nanoworms (16) and T7 phage extravasated and have enhanced accumulation in tumor cells upon iRGD coadministration. Therefore, we combined the tumor-penetrating peptide and contrast agents in our study. In addition, this method may also have the following additional advantages: (i) the combination does not involve SPIO surface modification, which may impair the biological characteristics or good compatibility of the particles, and (ii) the quantity or concentration of materials can easily be adjusted during the course of experiments.

In the present study, five different iron concentrations of SPIO were selected according to our previous preliminary experiment. Thorek and Tsourkas reported the appropriate iron oxide concentration as less than 50 *μ*g/mL in nonphagocytic cells [39]. Kobukai et al. reported that there were no registered dead dendritic cells treated with SPIO nanoparticles and PLL up to the 20 *μ*g/mL concentration threshold. Meanwhile, SPIO nanoparticles neither were toxic nor reduced the viability of the cells [37]. In our previous study, we found the appropriate iron concentration range for labeling panc-1 cells to be 21–42 *μ*g/mL. A similar result was reported by Boutry et al. Therefore, in the present study, a concentration lower than 21 *μ*g/mL was selected and ensured [40].

After 24 hours of incubation, differences in the SPIO uptake were found between the treatment and control groups. The control group had less pronounced signal and intracellular iron oxide content. However, with high iron concentrations (14.7–21 *μ*g/mL), the iRGD peptide effect reduced and was even inverse. This may be explained by the fact that the *α*v*β*3 integrins were saturated or phagocytosis became the predominant mechanism of nanoparticle uptake [31]. Another possible reason is that the saturation of *α*v*β*3 integrins may inhibit phagocytosis in the presence of high iron concentrations. Our study results show that the uptake and specific binding of our iRGD and SPIO to panc-1 cells in the treatment group were higher than those in the control group, which pointed to a receptor-mediated endocytosis mechanism with the appropriate iron and iRGD exposure. These findings were confirmed by light microscopy, transmission electron microscopy (TEM), and AAS.

A few points must be addressed for better understanding of the results and for improved design of more effective molecular labeling strategies. First, the present results suggest that a small quantity of iRGD peptides can optimally label cells* in vitro*. The longitudinal tracking of pancreatic cancer cells and further investigation* in vivo* using MR will be more challenging than* in vitro* studies. However, this study can be deemed a step towards* in vivo* tracking of pancreatic cancer cells with iRGD peptide and SPIO. Further investigation in animal tumor models remains a key goal of our ongoing studies.

In conclusion, our findings demonstrate that iRGD peptide affects the uptake of iron oxide during labeling of panc-1 cells. An appropriate iRGD peptide concentration can enhance the uptake of intracellular iron. The importance of this study lies in its description of a new potential strategy for pancreatic cancer imaging.
